# Pneumatosis Coli Formation via Counterperfusion Supersaturation in a Patient with Severe Diarrhea

**DOI:** 10.1155/2018/6931747

**Published:** 2018-08-05

**Authors:** Eric Vecchio, Sehrish Jamot, Jason Ferreira

**Affiliations:** Rhode Island Hospital and Brown University, USA

## Abstract

We present the case of an elderly male patient with known multiple myeloma who was hospitalized with profuse watery diarrhea and abdominal pain after a course of induction chemotherapy. Intestinal intramural gas was found on imaging and the diagnosis of pneumatosis intestinalis was confirmed by colonoscopy. We propose counterperfusion supersaturation as the etiology for this patient's pneumatosis coli via disruption of homeostasis between nitrogen and hydrogen normally present in the bowel. His condition was successfully treated with antidiarrheal medications and inhaled oxygen as well as intravenous hydration, and he eventually completed multiple myeloma directed chemotherapy with an excellent response. In this report, we discuss how clinicians can improve management of pneumatosis intestinalis by understanding the proposed pathophysiology.

## 1. Introduction

Pneumatosis intestinalis (PI) is a radiographic finding, which describes intestinal intramural air. It is frequently benign and does not typically require intervention [1, 2]. It is a rare condition affecting men and women equally and usually presents between the fourth and seventh decades of life [3]. Etiologies vary widely and range from idiopathic and benign to life threatening. The pathogenesis of this finding relies on the suspected origin of the intramural gas and the path it took to arrive in the bowel wall. Treatment is mainly supportive; however, additional therapy directed at the disease process responsible for producing pneumatosis intestinalis is typically required. In this report we present a case of pneumatosis intestinalis believed to be caused by counterperfusion supersaturation.

## 2. Case Report

An 86-year-old male with a past medical history significant for IgG lambda light chain multiple myeloma, congestive heart failure, and atrial fibrillation on anticoagulation presented for evaluation of abdominal pain, increasing abdominal distention, and inability to pass flatus or bowel movements for two days. Prior to his ileus-like symptoms, he experienced anorexia, rigors, and severe watery diarrhea for several days in correlation with the completion of his second cycle of Revlimid (lenalidomide), Velcade (bortezomib), and dexamethasone induction chemotherapy for multiple myeloma.

**Figure 1:**
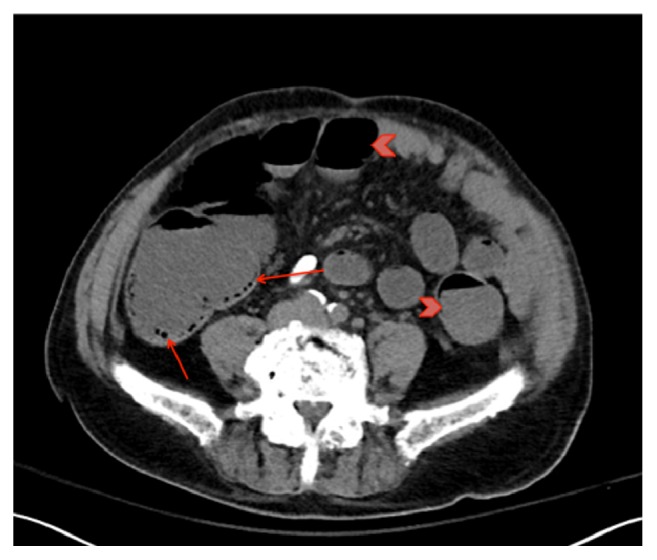
CT scan notable for colonic distention with submucosal and intraluminal gas (arrow), distended fluid-filled distal small bowel loops (arrowhead).

**Figure 2:**
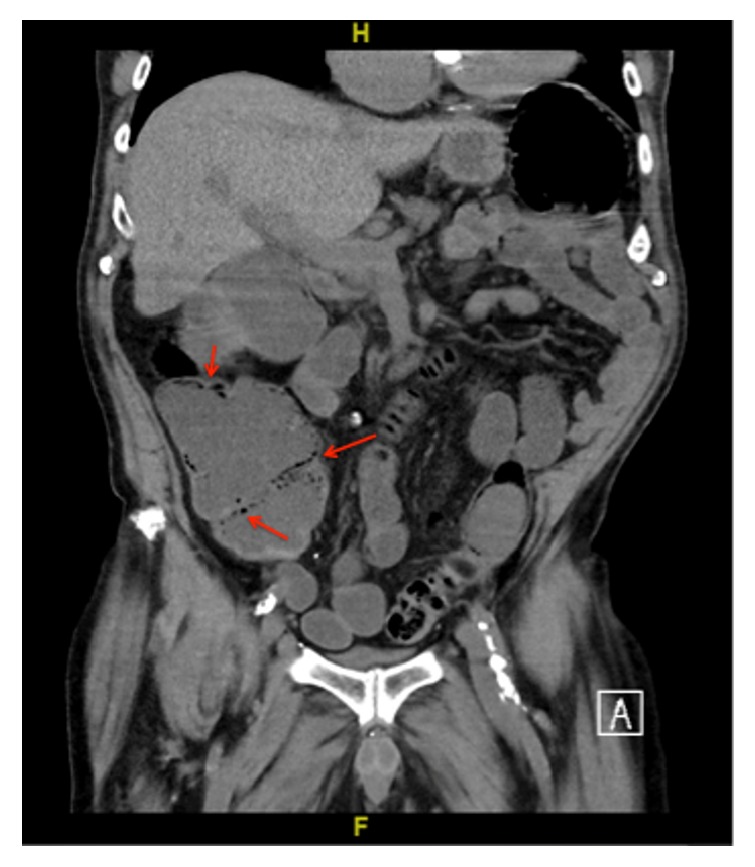
Coronal view with colonic wall thickening and submucosal and intraluminal air (arrow).

On presentation, he was afebrile and hypotensive to 72/42. Physical exam demonstrated a pale man with dry oral mucosa and a mildly tender, tympanitic abdomen without peritoneal signs. On laboratory examination he was found to have a white blood cell count of 4.6 x10^∧^9/L, hemoglobin of 8.1 g/dl, platelets of 15 x10^∧^9/L, creatinine of 2.35 mg/dL (baseline of 0.9 mg/dL), and lactic acid of 2.3 meq/L. A noncontrast abdominal computerized tomography (CT) scan was performed, which demonstrated diffuse colonic distention with submucosal and intraluminal gas, distended fluid-filled distal small bowel loops, and innumerable small submucosal lesions covering the surface of the colon (Figures 1 and 2). The clinical picture and data were interpreted as concern for an infectious colitis, but extensive stool testing including clostridium difficile toxin polymerase chain reaction, giardia antigen, cryptosporidium antigen, and stool cultures were all negative.

Initially, the diagnosis of bortezomib-induced ileus was entertained; however, the patient's diarrhea quickly resumed once he had been fluid resuscitated. He continued to have watery stools requiring rectal tube placement without improvement on piperacillin-tazobactam and metronidazole. The gastroenterology service was consulted to determine the etiology of his diarrhea and abdominal pain, at which point the diagnosis of pneumatosis coli was considered. Repeat CT scan with IV contrast was requested and revealed bowel wall thickening of the transverse and proximal descending colon as well as multiple mildly thickened mid and distal small bowel loops. Based on the updated CT findings, concern for underlying typhlitis arose and the patient was maintained on empiric antibiotic therapy. Due to continued voluminous watery diarrhea, hypotension requiring constant fluid administration, severe hypokalemia, and little to no improvement on antibiotics, the patient was prepped for colonoscopy for direct visualization of the abnormal appearing bowel on imaging.

**Figure 3:**
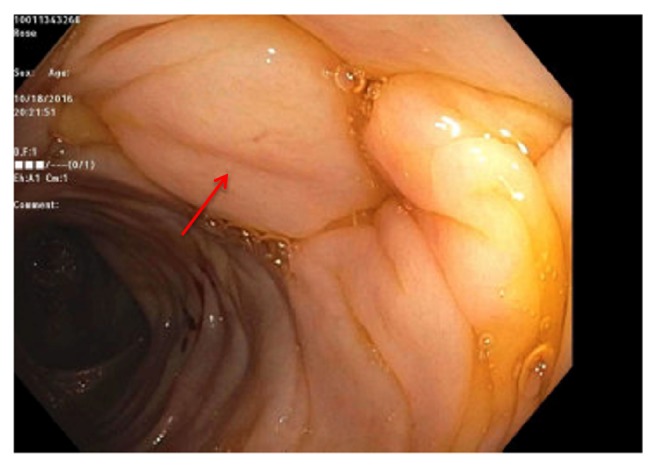
Pneumatosis coli of the transverse colon (arrow).

**Figure 4:**
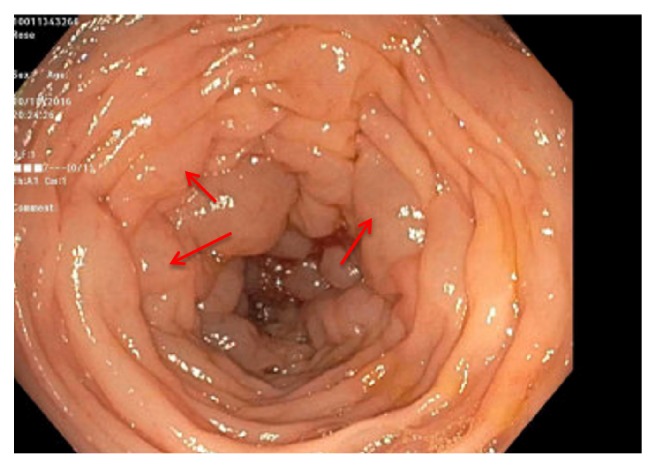
Pneumatosis coli of the descending colon (arrows).

**Figure 5:**
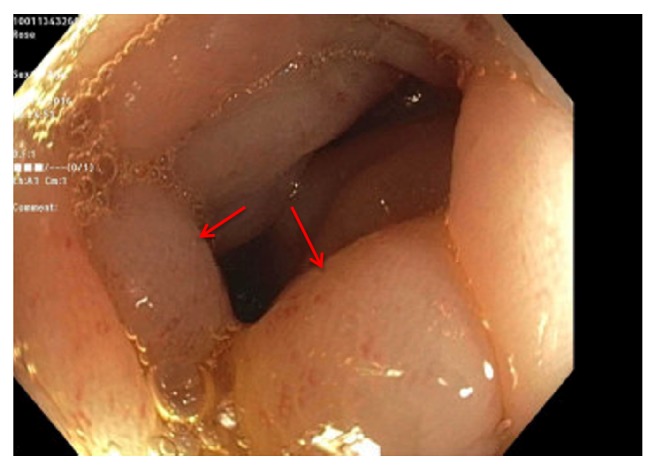
Pneumatosis coli of the sigmoid colon (arrows).

**Figure 6:**
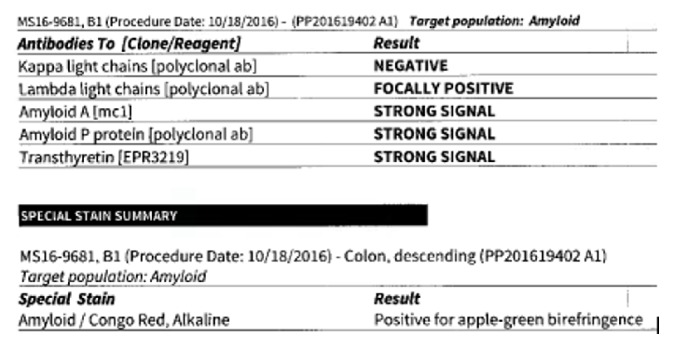
Pathology result of colonic biopsy confirming diagnosis of gastrointestinal amyloidosis.

On colonoscopy, the colonic mucosa was normal appearing except for the presence of diffuse intramural gas consistent with pneumatosis coli (Figures 3–5). Biopsies of the colonic lesions were obtained and sent for pathological evaluation. After confirming the diagnosis of pneumatosis coli on colonoscopy, the objective for patient care shifted to controlling stool output with antidiarrheal agents. Treatment was successful using a combination of diphenoxylate/atropine, cholestyramine, and loperamide. The surgical pathology of the colonic biopsies returned positive for apple green birefringence on Congo red stain consistent with gastrointestinal amyloidosis (Figure 6). The patient received continued treatment directed towards his multiple myeloma, which was thought to have caused the amyloidosis. The patient had a dramatic response to chemotherapy with resolution of his gastrointestinal symptoms.

## 3. Discussion

### 3.1. Pathophysiology

Pneumatosis intestinalis is the term used to describe pockets of gas found within the wall of the intestine. Determination of the origin of the deposited gas can help us understand the mechanisms by which pneumatosis intestinalis can occur. Gas can be found in the intestinal lumen due to ingestion of air, production by flora, production by invasive pathogenic gas-producing bacteria; can diffuse from nearby mesenteric blood vessels; or can be of pulmonary origin. One theorized mechanism for gas deposition within the luminal wall involves intraluminal air dissecting into the wall of the intestine via mucosal defects. Bacterial invasion of the intestinal wall by gas-producing organisms can also lead to deposition of gas in the submucosa of the intestine. Gas from ruptured lung parenchyma can dissect into the mediastinum to the retroperitoneum, from where it can eventually track along perivascular tissue planes to deposit in the wall of the bowel [2]. Finally, counterperfusion supersaturation is a mechanism by which gas diffusion gradients (particularly nitrogen and hydrogen) between the bowel and mesenteric blood vessels are disrupted. Specifically, gas cysts tend to occur in close proximity to blood vessels on the mesenteric border of the colon. Normally the colon lumen serves as a hydrogen source and a nitrogen sink, whereas the circulation is a hydrogen sink and a nitrogen source. This establishes a homeostatic exchange of the two gases. In scenarios where this steady state is perturbed, gas can deposit within the luminal wall and can be perpetuated until the ongoing pathology is properly addressed [4].

### 3.2. Presentation

Symptomatology of PI is largely nonspecific and is attributed to the underlying condition causing pneumatosis intestinalis. Frequently, patients present with diarrhea, abdominal pain, abdominal distention, nausea, vomiting, and weight loss if the diarrhea is protracted and mucous in the stool. Interestingly, PI is one of the differential diagnoses associated with sterile pneumoperitoneum [3].

### 3.3. Diagnosis

Diagnosis of pneumatosis intestinalis is largely imaging based. It can be diagnosed on X-ray, ultrasound, CT, and endoscopy among other modalities. Abdominal radiographs can demonstrate pneumatosis intestinalis in two-thirds of cases [5]. Ultrasonography can also be used to demonstrate pneumatosis intestinalis, which manifests itself as bright echoes found in the intestinal wall. The majority of studies investigating the presence of intraluminal gas relate to diagnosis of intestinal ischemia. Multidetector-row CT (MDCT) is the most commonly used method of imaging when it comes to sensitivity of detecting intramural gas [6]. In one small study, CT had a specificity of more than 95% for detecting the presence of intramural gas relating to intestinal ischemia. Even more important is the ability of MDCT to quickly image the entire abdomen, increasing the chances that the clinician will be able to identify the cause of the PI and rule out life threatening conditions.

### 3.4. Complications

Complications occur in about 3% of patients with pneumatosis intestinalis and include volvulus, bowel obstruction, gastrointestinal hemorrhage, and intestinal perforation manifested as pneumoperitoneum [3].

### 3.5. Management

In general, inhaled oxygen therapy has been cited as early as the 1970s as a treatment for PI [7, 8]. Studies are conclusive in demonstrating that oxygen is beneficial both at various concentrations and delivery routes from nasal cannula to hyperbaric oxygen therapy; however, the optimal dose has not been determined [9]. Oxygen therapy may directly improve symptoms by displacing gases found within the cysts with oxygen, which is subsequently metabolized causing resolution of the cysts [10]. Treatment should also be directed towards the underlying disease resulting in pneumatosis intestinalis as well as management of potential complications of pneumatosis including bowel necrosis, intestinal perforation, and peritonitis.

We believe the pneumatosis intestinalis in this patient formed via counterperfusion supersaturation for a number of reasons. First, his initial presentation with significant hypotension likely resulted in a disruption of the normal nitrogen and hydrogen homeostasis of the intestine by impairing diffusion of nitrogen from the blood to the gut due to low blood pressure. Additionally, chronic disruption of mesenteric vascular permeability as a result of amyloid vasculopathy may have contributed to the formation of pneumatosis intestinalis in this patient. Finally, there was likely an increase in hydrogen in the lumen of the intestine due to reduction in gut hydrogen absorption by intestinal bacteria as a result of antibiotic administration. Since the mucosa was not disrupted on colonoscopy, we do not believe the PI was formed as a result of amyloid deposition throughout the GI tract, despite the presence of amyloid on biopsy.

### 3.6. Conclusion

The differential for pneumatosis intestinalis is broad and includes both benign and life threatening conditions. Any disease state causing damage to the intestinal blood supply or disruption of the gut microbiome can potentially cause PI. Lack of knowledge of this condition and its proposed pathophysiology may limit timely diagnosis and as a result could cause patient harm. Specifically, patients are at risk for unnecessary surgery, extreme volume depletion, and electrolyte abnormalities due to uncontrolled diarrhea, side effects from antibiotic therapy, and increased hospital length of stay, all of which ultimately can result in poor outcomes and increased healthcare costs.

Pneumatosis intestinalis is a condition caused by the deposition of gas into the bowel wall. Air can make its way into the bowel by a number of pathologic mechanisms, all of which take into account either mucosal injury or pressure/diffusion gradients between body compartments. The diagnosis is imaging based and is best performed with MDCT scan due to its ability not only to identify the finding, but also to potentially reveal the underlying disease process. Treatment of pneumatosis intestinalis itself is supportive consisting of intravenous hydration and inhaled oxygen therapy. Clinical and laboratory evaluation to diagnose the underlying cause of the pneumatosis intestinalis is paramount and careful consideration is necessary prior to initiating aggressive interventions.

## Data Availability

Previously reported data were used to support this study and are available below. These prior studies (and datasets) are cited at relevant places within the text as [1–10].
